# Direct breastfeeding during the first week after NICU discharge among preterm infants: prevalence and associated factors

**DOI:** 10.3389/fped.2026.2002068

**Published:** 2026-10-30

**Authors:** Yuan-yuan Zhang, Rong Liu, Pu Zhao, Li-Li Chen, Xue-lei Yin, Wenting Zheng, Hong Zhang, Li-Jin Zhao

**Affiliations:** 1 The First People's Hospital of Taicang, Taicang Affiliated Hospital of Soochow University, Taicang, China; 2 School of Medicine, Tongji University, Shanghai, China; 3 Shanghai First Maternity and Infant Hospital, School of Medicine, Tongji University, Shanghai, China

**Keywords:** associated factors, breastfeeding self-efficacy, direct breastfeeding, neonatal intensive care unit, preterm infants

## Abstract

**Background:**

Direct breastfeeding (DBF) offers nutritional and immunological benefits and supports the coordination of sucking, swallowing, and breathing in preterm infants, yet many preterm infants do not feed directly at the breast around the time of neonatal intensive care unit (NICU) discharge. This study aimed to describe the proportion of preterm infants who fed directly at the breast during the first 7 days after NICU discharge and to identify factors associated with this outcome.

**Methods:**

This was a multicenter prospective cohort study conducted in the NICUs of two tertiary hospitals in China. Using consecutive convenience sampling, we enrolled 421 preterm infant-parent units between July 2025 and July 2026. During hospitalization, mothers independently completed a general information questionnaire, the Breastfeeding Self-Efficacy Scale-Short Form (BSES-SF), the Edinburgh Postnatal Depression Scale (EPDS), and the Self-Rating Anxiety Scale (SAS), while fathers completed the Father's Breastfeeding Self-Efficacy Scale-Short Form (FBSES-SF). Feeding status was assessed at the 1-week post-discharge follow-up and classified into three mutually exclusive categories (exclusive DBF, partial DBF, and no DBF), with exclusive and partial DBF combined into a derived binary outcome, any DBF. Binary logistic regression was used to identify factors associated with any DBF, with study center retained as a fixed effect.

**Results:**

Of the 421 preterm infants, 215 (51.07%) had any DBF during the first 7 days after NICU discharge. Of these, 76 (18.05%) were exclusively directly breastfed, and 139 (33.02%) were partially directly breastfed; the remaining 206 (48.93%) had no DBF. In the adjusted binary logistic regression model (*n* = 404; 213 with any DBF and 191 with no DBF), the following variables were associated with any DBF (adjusted odds ratio [aOR], 95% confidence interval [CI]): oropharyngeal colostrum administration, ≥1 vs. 0 sessions (aOR 5.58, 95% CI 2.48–12.58); severity of illness (overall *P* < 0.001), grade 3 vs. grade 1 (aOR 0.09, 95% CI 0.03–0.24) and grade ≥4 vs. grade 1 (aOR 0.02, 95% CI 0.01–0.07); DBF during hospitalization, ≥1 vs. 0 sessions (aOR 5.41, 95% CI 2.34–12.50); maternal educational level, bachelor's degree or above vs. junior college or below (aOR 4.78, 95% CI 1.97–11.58); monthly household income (overall *P* = 0.008), 10,000–19,999 CNY vs. < 10,000 CNY (aOR 4.16, 95% CI 1.54–11.19) and ≥20,000 CNY vs. < 10,000 CNY (aOR 4.35, 95% CI 1.30–14.54); anxiety score, per 1-point increase (aOR 0.90, 95% CI 0.85–0.96); and paternal breastfeeding self-efficacy, per 1-point increase (aOR 1.07, 95% CI 1.04–1.10). Severity of illness grade 2 vs. grade 1 and study center did not reach statistical significance in the adjusted model.

**Conclusions:**

About half of the preterm infants in this cohort fed directly at the breast during the first 7 days after NICU discharge. In this observational study, receipt of oropharyngeal colostrum administration, DBF during hospitalization, higher maternal educational level, higher monthly household income, and higher paternal breastfeeding self-efficacy were associated with higher odds of any DBF, whereas greater severity of illness and higher maternal anxiety score were associated with lower odds of any DBF. These associations do not establish causal effects and require confirmation in interventional studies before they are used to guide care.

## 1 Introduction

With advances in medical care, the survival rate of preterm infants has risen steadily. However, the growth, development, and nutritional status of preterm infants continue to lag behind those of term infants of the same postnatal age, and this disadvantage can compromise their long-term health (1). For term infants, feeding directly at the breast is a natural and readily achieved practice, although the proportion of term infants who establish feeding at the breast without any difficulty varies across settings (2). For preterm infants, in contrast, DBF is a considerable challenge. Immature coordination of sucking, swallowing, and breathing, together with mother-infant separation during hospitalization, creates major obstacles to DBF, and many preterm infants still cannot feed directly at the breast by the time they are discharged. This not only threatens the continuation of breastfeeding after discharge but may also deprive infants of the sucking practice needed for oral motor development. Feeding directly at the breast combines nutritional and immunological value and supports the coordination of sucking, swallowing, and breathing (3), which may reduce the risk of oxygen desaturation and bradycardia and shorten the transition to full oral feeding. although expressed milk retains most macronutrients and bioactive components, feeding directly at the breast is the only mode that delivers milk freshly expressed and undegraded and that simultaneously provides the sensory, interactive, and physiological regulation that accompanies nursing (4). Human milk feeding, whether at the breast or by bottle, supports gut microbial development (5); direct feeding at the breast additionally avoids the mismatch between the timing of milk secretion and the timing of feeding, better supports the development of sleep-wake cycles, and provides sensory, verbal, and non-verbal (emotional) communication (6). Reported rates of DBF during hospitalization range from 10% to 52.4% in developed countries (7, 8). In China, the semi-closed NICU model means that preterm infants are fed mainly bottled expressed breast milk, with very little DBF during hospitalization, and few data on DBF during hospitalization or in the early post-discharge period have been reported. The World Health Organization recommends exclusive breastfeeding for infants during the first 6 months of life, and the World Health Assembly has set a global target of raising the exclusive breastfeeding rate at 6 months to at least 50% by 2025 (9); in practice, however, only 10.4% of preterm infants are exclusively breastfed at 6 months of age (10). Although the importance of DBF is increasingly recognized, research specifically addressing DBF in the early post-discharge period among preterm infants is limited (11). Existing Chinese data have addressed post-NICU-discharge exclusive breastfeeding and its duration in first-time mothers of preterm infants (10), but direct feeding at the breast has rarely been examined in this population. A 2022 longitudinal cohort study conducted in Shanghai examined exclusive breastfeeding after NICU discharge (10); however, because it did not specifically assess direct feeding at the breast, the evidence base concerning DBF during the early post-discharge period in Chinese preterm infants remains limited. Clinical assessment and intervention strategies for DBF therefore still lack a targeted evidence base. Existing studies suggest that socioeconomic status, maternal age, and marital status may be associated with the successful establishment of DBF in the NICU (12–15), but whether these factors are relevant to preterm infants in China remains undetermined. Because whether DBF can be established during the early post-discharge period is closely related to the continuation of breastfeeding and to the development of oral motor function, clarifying the proportion of preterm infants who feed directly at the breast during this period in Chinese NICUs, and the factors associated with this outcome, is particularly important. This study therefore aimed to describe the proportion of preterm infants feeding directly at the breast during the first 7 days after NICU discharge and to examine factors associated with this outcome, in order to inform future intervention development.

## 2 Materials and methods

### 2.1 Study design and participants

This was a multicenter prospective observational cohort study conducted in the NICUs of two tertiary hospitals in China: Shanghai First Maternity and Infant Hospital (Shanghai) and Taicang First People's Hospital (Taicang, Jiangsu Province). A consecutive convenience sampling strategy was used to recruit preterm infant-parent units between July 2025 and July 2026. All eligible families admitted during the recruitment period were approached in sequence until the target sample size was reached. The temporal sequence of the study was as follows: (1) screening and enrollment of eligible preterm infants within 72 h of NICU admission; (2) collection of infant clinical and hospitalization-related variables from the medical record; (3) administration of the BSES-SF, FBSES-SF, EPDS, and SAS during hospitalization, before discharge; (4) discharge of the infant home; (5) assessment of breastfeeding status at the 1-week post-discharge follow-up; and (6) completion of the analytic dataset. All exposure and covariate measurements therefore preceded the assessment of the primary outcome. The inclusion criteria for preterm infants were: (1) gestational age at birth of 28 to <37 weeks; (2) birth weight <2,500 g; and (3) transfer to the NICU immediately after birth. The exclusion criteria were: (1) inability to tolerate enteral nutrition after 1 week of hospitalization; (2) severe congenital disease requiring surgery; and (3) conditions precluding breastfeeding, such as breast milk intolerance, necrotizing enterocolitis, or breast milk jaundice. The inclusion criteria for parents were: (1) father aged ≥22 years and mother aged ≥20 years; (2) clear consciousness, ability to communicate verbally and in writing, and adequate reading and comprehension ability; and (3) provision of informed consent and willingness to complete the questionnaire survey and follow-up. The exclusion criteria for parents were: (1) maternal contraindication to breastfeeding (e.g., HIV infection) (16); and (2) a history of psychiatric illness. Study center was recorded for each participant, and the number of infants recruited at each hospital is reported in the Results. A total of 446 preterm infants were screened and assessed for eligibility during the recruitment period. Ten infants were excluded: three had a severe congenital disease requiring surgery, three had necrotizing enterocolitis, and four had breast milk jaundice. The remaining 436 eligible families were approached, of whom 15 declined to participate because they were unwilling to complete the questionnaire or the 1-week follow-up. A total of 421 preterm infant-parent units were therefore enrolled, and all 421 completed both the discharge assessment and the 1-week post-discharge follow-up (215 with any DBF and 206 with no DBF). All 421 were included in the univariate analysis; 404 of the 421 had complete data for every variable in the multivariable model and entered the adjusted regression analysis (213 with any DBF and 191 with no DBF). No additional selection was applied beyond the eligibility criteria described below; eligible families admitted during the recruitment period were approached consecutively until the target sample size was reached. The participant flow is summarized in Figure 1. All parents took part voluntarily, and two separate consents were obtained. Each mother and father provided written informed consent for their own participation in the questionnaire survey, including the psychological and breastfeeding-related measures, and the parents or legal guardians of each infant additionally provided written informed consent for the use of that infant's clinical and hospitalization data. The study was approved by the Medical Ethics Committee of Shanghai First Maternity and Infant Hospital (approval no. 2025 Ethics Review No. 299) and by the Medical Ethics Committee of Taicang First People's Hospital (approval no. 2026-1w-014). For both hospitals, data collection was initiated only after ethics approval had been granted by the respective institutional ethics committees.

**Figure 1:**
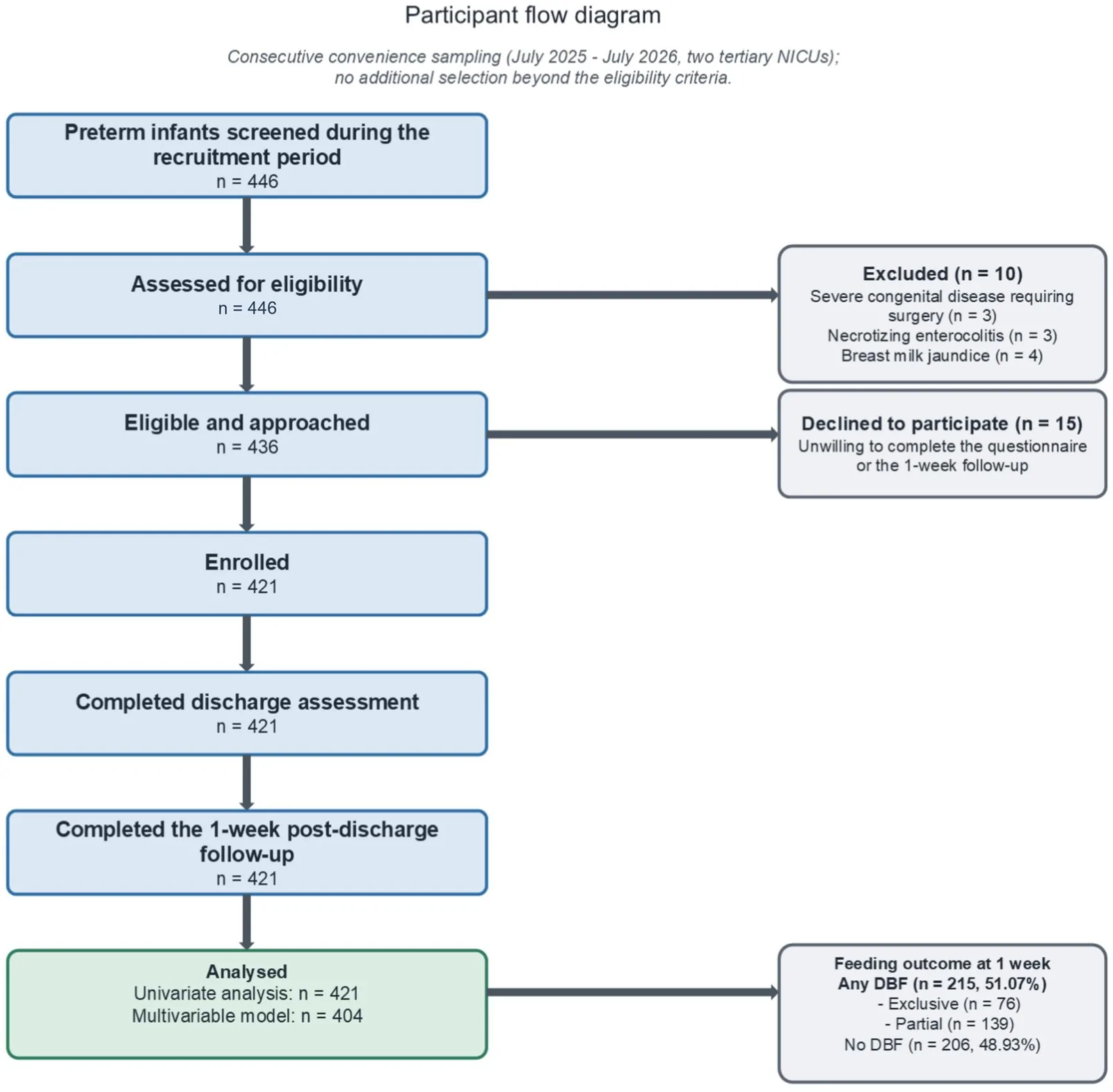
Flow of participants through the study. Of the 421 enrolled infant-parent units, all completed the discharge assessment and the 1-week post-discharge follow-up and were included in the univariate analysis; 404 had complete data for every variable in the multivariable model and entered the adjusted analysis (213 with any DBF and 191 with no DBF). DBF, direct breastfeeding; NICU, neonatal intensive care unit.

### 2.2 Measurements

#### 2.2.1 General information questionnaire

The questionnaire was developed by the investigators after a literature review and expert consultation. It collected general information about the mother (age, educational level, monthly household income, occupation, and so on), information about the infant (sex, gestational age, birth weight, mode of delivery, severity of illness, duration of mechanical ventilation, duration of CPAP, days with an indwelling gastric tube, gestational age at first oral feeding, time to regain birth weight, and total length of hospital stay), and information about interventions during hospitalization (oropharyngeal colostrum administration, kangaroo mother care, DBF during hospitalization, family-centered ward, time to initiate milk expression after delivery, use of a breast pump within 6 h after delivery, frequency of breastfeeding or pumping within 48 h after delivery, and breast massage within 24 h after delivery). Severity of illness was graded using the Neonatal Medical Index (NMI) (17, 18), which is scored after NICU admission from birth weight, respiratory support, neurological findings, and other major complications. The NMI classifies infants from grade I (least severe) to grade V (most severe); the full grading criteria are provided in Supplementary Table 1. For the analysis, grades IV and V were combined into a single category (grade ≥4), because few infants were classified as grade V. Oropharyngeal colostrum administration was defined as the instillation of freshly expressed colostrum (0.2–0.5 mL per session, every 3–4 h, according to the NICU protocol) onto the oral mucosa, followed by oropharyngeal massage, and was dichotomized as none versus ≥1 session because the number of sessions recorded in the questionnaire was not sufficiently granular to support finer categories. A DBF session during hospitalization was defined as the infant being put to the breast in the presence of a nurse for at least 5 min, regardless of the volume of milk transferred, and the variable was likewise dichotomized as none versus ≥1 session. Kangaroo mother care was defined as skin-to-skin contact for at least 1 h per day; family-centered ward admission was defined as the mother being admitted to the same room as the infant for rooming-in care.

#### 2.2.2 Breastfeeding self-efficacy scale-short form (BSES-SF)

The BSES-SF was developed by Dennis et al. in 2003 and translated into Chinese and validated by Liu et al. (19) in 2016; the Cronbach's alpha of the Chinese version is 0.927. The scale contains 14 items rated on a 5-point Likert scale, from “not at all confident” to “very confident,” scored from 1 to 5. Total scores range from 14 to 70, with higher scores indicating greater maternal breastfeeding self-efficacy. In this study, the BSES-SF was administered to mothers during hospitalization, within 72 h before the infant was discharged, and this single measurement was used in the regression analysis.

#### 2.2.3 Father support breastfeeding self-efficacy scale-short form (FBSES-SF)

The FBSES-SF was adapted from the Breastfeeding Self-Efficacy Scale by Dennis et al. (20) and assesses fathers’ confidence in supporting their partner's breastfeeding. Zhou et al. (21) translated and validated the scale in China in 2020, reporting Cronbach's alpha values of 0.902–0.938. The scale contains 14 items rated on a 5-point Likert scale, with total scores ranging from 14 to 70; higher scores indicate greater paternal confidence in supporting breastfeeding. The FBSES-SF was administered to fathers during hospitalization, within 72 h before the infant was discharged.

#### 2.2.4 Edinburgh postnatal depression scale (EPDS)

The EPDS was developed by Cox et al. and translated into Chinese by Lee et al. (22) in 1998. It contains 10 items covering mood, anhedonia, self-blame, anxiety, fear, insomnia, coping ability, sadness, crying, and self-harm, each rated on a 4-point Likert scale (0 = never, 1 = occasionally, 2 = often, 3 = always). Total scores range from 0 to 30, with higher scores indicating more severe depressive symptoms. A score of <10 indicates no depression, whereas a score of ≥10 indicates probable depression. The Cronbach's alpha of the Chinese version is 0.73. The EPDS was administered to mothers during hospitalization, within 72 h before the infant was discharged, and raw item scores were summed to give the total score used in the analyses.

#### 2.2.5 Self-rating anxiety scale (SAS)

The SAS was developed by Zung (23) in 1971 and contains 20 items rated on a 4-point Likert scale. Raw scores are summed and then multiplied by 1.25 to give the standardized score. A standardized score of <50 indicates no anxiety, 50–59 mild anxiety, 60–69 moderate anxiety, and ≥70 severe anxiety. The raw score was used in all analyses presented here. Cronbach's alpha for the scale in the present study was 0.879. The SAS was administered to mothers during hospitalization, within 72 h before the infant was discharged.

### 2.3 Data collection and definitions of the feeding outcome

The primary outcome was any direct breastfeeding during the first 7 days after NICU discharge. DBF was defined as the infant being put to the breast, irrespective of the volume of milk transferred or the number of feeds; this definition was based on a reported feeding pattern rather than a single feeding episode. Because the definition did not require demonstrated milk transfer, a feeding episode was not distinguished from an attempt without effective milk transfer, and the outcome therefore reflects the mother's report of whether the infant was put to the breast during that week. The outcome was operationalized into three mutually exclusive feeding categories: (1) exclusive DBF, defined as all oral feeds during that period being taken directly at the breast, with no bottle feeds of expressed human milk or formula; (2) partial DBF, defined as a mixture of direct feeding at the breast and bottle feeding (expressed human milk or formula) during that period; and (3) no DBF, defined as no episode of feeding directly at the breast during that period, irrespective of whether human milk or formula was given and irrespective of the route by which it was given. From these three categories a derived binary outcome, any DBF, was formed by combining exclusive DBF and partial DBF, so that any DBF represents at least one episode of feeding directly at the breast at any time during the first 7 days after NICU discharge. Exclusive DBF and partial DBF were combined into a single binary outcome, any DBF, because the aim was to compare infants who fed directly at the breast at all with those who did not: any feeding directly at the breast, even partial, requires the infant to coordinate sucking, swallowing, and breathing at the breast and is therefore the behavior of interest for breastfeeding promotion, whereas exclusive and partial DBF are reported separately as descriptive outcomes. The category is deliberately labeled no DBF rather than bottle feeding, because milk type (human milk versus formula) and feeding route (bottle versus nasogastric tube) were not recorded separately in this study; infants in this category received no direct breastfeeding, but the type and route of the milk they received are not distinguished here. Feeding status was reported by the mother at the 1-week post-discharge follow-up and was cross-checked against the feeding record kept by the parents and, where the infant attended an outpatient visit, against the clinical record. Because assessment at the 1-week follow-up covers the whole post-discharge week, the outcome is reported throughout as any DBF during the first 7 days after NICU discharge; it does not represent a point prevalence measured on the day of discharge. Data were collected at three time points. (1) During hospitalization: infant clinical variables were abstracted from the medical record, and hospitalization-related feeding and care practices were recorded on the ward; the BSES-SF, FBSES-SF, EPDS, and SAS were administered to mothers and fathers within 72 h before discharge. (2) At discharge (face to face): maternal demographic, socioeconomic, and family-structure variables were recorded. (3) At the 1-week post-discharge follow-up (outpatient visit or telephone interview): the feeding outcome was ascertained. Before data collection, uniformly trained investigators used standardized instructions to explain the purpose, significance, and requirements of the survey to participants. During hospitalization and at discharge, mothers and fathers completed the questionnaires independently; the questionnaires were collected on site and checked for completeness. An item was considered “incorrect or missing” only when it had been unintentionally omitted or when two mutually exclusive options had both been selected; in these cases the respondent was asked to complete or clarify the item, and the item was never revised on the basis of the investigator's own judgment. Because the questionnaire was completed before discharge, the investigator had no knowledge of the post-discharge feeding outcome at the time of checking. No items were missing among the 421 returned questionnaires after on-site checking, and follow-up was complete: all 421 enrolled infant-parent units (100%) completed the 1-week post-discharge follow-up. A small number of clinical variables were incomplete in the medical record, however. Ventricular septal defect or patent ductus arteriosus was missing for 14 infants (3.33%), duration of CPAP and the frequency of breastfeeding or pumping within 48 h after delivery were each missing for 2 infants (0.48%), and each of the remaining variables with missing data was missing for 1 infant (0.24%). The four scale total scores (BSES-SF, FBSES-SF, EPDS, and SAS) were complete for all 421 mothers and fathers. The number of infants with available data therefore differs between variables in Table 1 and the percentage denominators are not uniformly 421; the denominator used for each variable is given in the Table 1 note. The number of missing values for each variable, overall and by feeding group, is reported in Supplementary Table 5. No imputation was performed, each analysis used all available data, and all 421 infant-parent units were retained in the univariate analysis; 404 of the 421 infant-parent units had complete data for every variable in the multivariable model and entered the complete-case regression analysis. The 100% valid questionnaire response rate refers to questionnaire completeness only and should not be interpreted as evidence of complete data for variables collected outside the questionnaire.

**Table 1:** Univariate analysis of factors associated with any direct breastfeeding during the first 7 days after NICU discharge among preterm infants (unadjusted estimates). Percentages are calculated on the number of infants with available data for each variable, so denominators differ between variables; the denominator is given in the table note for every variable for which it differs from 421.

Variable	Category	Total (*n* = 421)	No DBF (*n* = 206)	DBF (*n* = 215)	Statistic	*P*
Total length of hospital stay (days)	–	12.00 (8.00, 22.00)	19.00 (11.00, 31.00)	9.00 (7.00, 13.00)	Z = 9.087	<0.001#
Duration of CPAP (days)	–	3.00 (0.00, 6.00)	5.00 (3.00, 11.00)	1.00 (0.00, 4.00)	Z = 8.528	<0.001#
Gestational age at first oral feeding (weeks)	–	35.00 (34.00, 35.00)	34.00 (34.00, 35.00)	35.00 (34.00, 35.00)	Z = −4.058	<0.001#
Time to regain birth weight (days)	–	10.00 (8.00, 13.00)	12.00 (10.00, 14.00)	8.00 (7.00, 11.00)	Z = 8.842	<0.001#
Days with indwelling gastric tube (days)	–	5.00 (1.00, 15.00)	11.00 (5.00, 23.00)	3.00 (0.00, 7.00)	Z = 8.565	<0.001#
Sex					*χ*^2^ = 1.043	0.307
	Male	236 (56.19)	110 (53.66)	126 (58.60)		
	Female	184 (43.81)	95 (46.34)	89 (41.40)		
Mode of delivery					χ^2^ = 3.269	0.071
	Vaginal delivery	98 (23.33)	40 (19.51)	58 (26.98)		
	Cesarean section	322 (76.67)	165 (80.49)	157 (73.02)		
Ventricular septal defect or patent ductus arteriosus					χ^2^ = 11.623	<0.001
	Yes	132 (32.43)	79 (40.72)	53 (24.88)		
	No	275 (67.57)	115 (59.28)	160 (75.12)		
Oropharyngeal colostrum administration					χ^2^ = 113.08	<0.001
	None	194 (46.19)	149 (72.68)	45 (20.93)		
	≥1 session	226 (53.81)	56 (27.32)	170 (79.07)		
Birth weight (g)	–	2,130.0 (1,800.0, 2,345.0)	1,895.0 (1,580.0, 2,182.0)	2,255.0 (2,075.0, 2,530.0)	Z = −9.321	<0.001#
Severity of illness					χ^2^ = 98.346	<0.001
	Grade 1	125 (29.76)	34 (16.59)	91 (42.33)		
	Grade 2	84 (20.00)	18 (8.78)	66 (30.70)		
	Grade 3	157 (37.38)	109 (53.17)	48 (22.33)		
	Grade ≥4	54 (12.86)	44 (21.46)	10 (4.65)		
Gestational age at birth (weeks)	–	34.00 (33.00, 35.00)	33.00 (32.00, 34.00)	35.00 (34.00, 35.00)	Z = −8.325	<0.001#
Admission to family-centered ward					χ^2^ = 72.020	<0.001
	Yes	189 (45.00)	49 (23.90)	140 (65.12)		
	No	231 (55.00)	156 (76.10)	75 (34.88)		
DBF during hospitalization					χ^2^ = 115.25	<0.001
	None	230 (54.76)	167 (81.46)	63 (29.30)		
	≥1 session	190 (45.24)	38 (18.54)	152 (70.70)		
Kangaroo mother care					χ^2^ = 131.08	<0.001
	None	184 (43.81)	148 (72.20)	36 (16.74)		
	≥1 session	236 (56.19)	57 (27.80)	179 (83.26)		
Type of medical insurance					χ^2^ = 30.726	<0.001
	Urban resident basic medical insurance	194 (46.19)	123 (60.00)	71 (33.02)		
	Urban employee basic medical insurance	226 (53.81)	82 (40.00)	144 (66.98)		
Educational level					χ^2^ = 132.46	<0.001
	Junior college or below	199 (47.38)	156 (76.10)	43 (20.00)		
	Bachelor's degree or above	221 (52.62)	49 (23.90)	172 (80.00)		
Monthly household income (CNY)					χ^2^ = 76.710	<0.001
	<10,000	186 (44.29)	132 (64.39)	54 (25.12)		
	10,000–19,999	136 (32.38)	55 (26.83)	81 (37.67)		
	≥20,000	98 (23.33)	18 (8.78)	80 (37.21)		
Time to initiate milk expression after delivery					χ^2^ = 155.63	<0.001
	Within 6 h	175 (41.67)	25 (12.20)	150 (69.77)		
	Within 12 h	153 (36.43)	99 (48.29)	54 (25.12)		
	>12 h	92 (21.90)	81 (39.51)	11 (5.12)		
Breast pump use within 6 h after delivery					χ^2^ = 44.761	<0.001
	Yes	283 (67.38)	106 (51.71)	177 (82.33)		
	No	137 (32.62)	99 (48.29)	38 (17.67)		
Frequency of breastfeeding or pumping within 48 h after delivery (times)	–	10.00 (6.00, 10.00)	6.00 (4.00, 8.50)	10.00 (10.00, 18.00)	Z = −12.80	<0.001#
Breast massage within 24 h after delivery					χ^2^ = 24.895	<0.001
	Yes	347 (82.62)	150 (73.17)	197 (91.63)		
	No	73 (17.38)	55 (26.83)	18 (8.37)		
Depression score (points)	–	8.37 ± 3.09	9.29 ± 3.27	7.49 ± 2.63	t = 6.235	<0.001†
Anxiety score (points)	–	26.58 ± 8.20	29.69 ± 9.97	23.60 ± 4.30	t = 8.196	<0.001†
Maternal breastfeeding self-efficacy (points)	–	44.90 ± 17.33	31.80 ± 9.92	57.46 ± 13.09	t = 22.595	<0.001†
Paternal breastfeeding self-efficacy (points)	–	45.70 ± 17.73	34.09 ± 12.36	56.82 ± 14.72	t = 17.123	<0.001†
Study center					χ2 = 5.904	0.015
	Center 1	253 (60.10)	136 (66.02)	117 (54.42)		
	Center 2	168 (39.90)	70 (33.98)	98 (45.58)		
Mechanical ventilation					χ2 = 0.121	0.728
	No	351 (83.37)	170 (82.52)	181 (84.19)		
	Yes	69 (16.39)	35 (16.99)	34 (15.81)		

Categorical variables were compared using Pearson's χ2 test; # rank-sum test; † independent-samples t-test. The denominator is 421 for the continuous variables and for the total sample; because data were missing for some categorical variables, the denominator is 420 for most categorical variables, 419 for duration of CPAP and for the frequency of breastfeeding or pumping within 48 h after delivery, and 407 for ventricular septal defect or patent ductus arteriosus. Missing values were not imputed, and each comparison used all available data. DBF, direct breastfeeding; CPAP, continuous positive airway pressure; SD, standard deviation; IQR, interquartile range.

### 2.4 Statistical analysis

Data were analyzed using SPSS 26.0. Normally distributed continuous variables were expressed as mean ± standard deviation and compared between groups using independent-samples t-tests; non-normally distributed continuous variables were expressed as median and interquartile range [M (P25, P75)] and compared using Mann–Whitney U tests. Categorical variables were expressed as frequencies and percentages and compared using the chi-squared test. Two-sided *P* < 0.05 was considered statistically significant. Sample-size justification. The planned sample size was based on the number of events rather than on the total number of participants, with an anticipated proportion of any DBF of approximately 50% used for planning. The 27 variables initially considered corresponded to 31 slope parameters, and the 23 candidate variables corresponded to 27 slope parameters; each model also contained one intercept. Of the 421 infant-parent units with complete follow-up, 404 had complete data for all variables in the model and entered the complete-case regression analysis, of whom 213 had any DBF and 191 had no DBF. The final adjusted model contained eight variables (11 slope parameters plus one intercept), so that each slope parameter corresponded to 19.36 outcome events and 17.36 non-events. Judged by the parameter burden of the final model, the analysis therefore met the conventional events-per-parameter criterion and provided reasonable information for the exploratory estimation of associations. This ratio applies only to the final model and does not validate the variable-selection procedure itself; the full model contained 28 variables (approximately 32 slope parameters plus the intercept), so the events-per-parameter ratio during selection was substantially lower, which is why the selection process must be interpreted as exploratory. The initially reported target of 297–446 participants was derived from a rule of 10–15 participants per variable applied to the 27 initially considered variables, with a further 10% allowance for invalid questionnaires. Variable selection. Twenty-seven independent variables were considered *a priori* on the basis of the literature and clinical reasoning, and all were treated as candidate predictors; none was pre-specified as an *a priori* confounder. In the original analysis, three of these variables - duration of mechanical ventilation, mode of delivery, and infant sex - were excluded before modelling because they did not differ between the feeding groups in the univariate comparison (*P* > 0.05), and one - breast pump use within 6 h after delivery - had been inadvertently omitted. The 27 variables originally considered, the 23 that were eligible for the multivariable model, and the four that were excluded before the original modelling, together with the reason for each exclusion, are listed in Supplementary Table 2. The complete results of the full multivariable model, before the variables with a Wald *P* < 0.05 were retained and refitted, are given in Supplementary Table 3. In the revised analysis, all variables examined in the univariate analysis were entered simultaneously into a full multivariable model together with study center, which was retained as a fixed effect, giving a full model that contained 28 variables. Variables with a Wald *P* < 0.05 in this full model were retained in a single step and refitted to give the final adjusted model; study center was retained as a fixed effect regardless of its *P* value. Continuous variables were entered on their original scale and categorical variables were entered as dummy variables. This data-driven approach was used because the study was exploratory and aimed at identifying associations rather than estimating a prespecified causal effect; the model is therefore associational/explanatory rather than predictive, and the variables retained should not be interpreted as representing all independent determinants of DBF. Model diagnostics and assumptions. Multicollinearity, functional form, and calibration were assessed and are reported separately, because they address different properties of the model. The linearity of the relationship between each continuous predictor (anxiety score and paternal breastfeeding self-efficacy) and the log odds of any DBF was assessed with the Box-Tidwell test. Multicollinearity was assessed with the variance inflation factor (VIF). Calibration was assessed with the Hosmer-Lemeshow test, and overall predictive performance with the Brier score. Study center and family-level clustering. Study center was included as a fixed effect in the multivariable model. Of the 421 participants, 253 were recruited at center 1 and 168 at center 2. The proportion of infants with any DBF differed between the two centers (117/253, 46.25% at center 1 vs. 98/168, 58.33% at center 2; chi-squared = 5.904, *P* = 0.015). Gestational age at birth, birth weight, severity of illness, DBF during hospitalization, admission to a family-centred ward, the anxiety score, and the paternal breastfeeding self-efficacy score did not differ significantly between the two centers, whereas the proportion of infants who received oropharyngeal colostrum administration (121/253, 47.83% vs. 105/168, 62.50%; chi-squared = 8.508, *P* = 0.004) and the proportion who received kangaroo mother care (130/253, 51.38% vs. 106/168, 63.10%; chi-squared = 5.973, *P* = 0.015) were higher at center 2. The feeding distribution by center is reported in Table 1, and the association between center and the outcome after adjustment for the other variables is reported in Table 3. A full comparison of baseline and care-practice characteristics between the two centers is given in Supplementary Table 4. With regard to family structure, only one infant per family was included in the analysis; infants from multiple births were not analyzed as separate observations, so no within-family correlation was present in the analytic dataset. Conceptual role of DBF during hospitalization. DBF during hospitalization was pre-specified as one of the covariates included in the multivariable model on the basis of its clinical relevance, without asserting a specific causal role (confounder versus mediator). The study was designed as an association analysis rather than a formal causal-inference study, and no formal mediation analysis was performed, because the objective was to describe adjusted associations rather than to estimate causal total effects.

## 3 Results

### 3.1 Status of direct breastfeeding during the first week after NICU discharge and univariate analysis

At the 1-week post-discharge follow-up, during which feeding status over the preceding week was ascertained, 215 of the 421 preterm infants (51.07%) had any DBF and 206 (48.93%) had no DBF; among those with any DBF, 76 (18.05%) fed exclusively at the breast and 139 (33.02%) fed partly at the breast (Table 1). Statistically significant differences between the any-DBF and no-DBF groups were observed for total length of hospital stay, duration of CPAP, gestational age at first oral feeding, time to regain birth weight, days with an indwelling gastric tube, birth weight, gestational age at birth, ventricular septal defect or patent ductus arteriosus, oropharyngeal colostrum administration, severity of illness, admission to a family-centered ward, DBF during hospitalization, kangaroo mother care, type of medical insurance, educational level, monthly household income, time to initiate milk expression after delivery, breast pump use within 6 h after delivery, frequency of breastfeeding or pumping within 48 h after delivery, breast massage within 24 h after delivery, depression score, anxiety score, maternal breastfeeding self-efficacy, paternal breastfeeding self-efficacy, and study center (all *P* < 0.05). No statistically significant differences were found for duration of mechanical ventilation (use vs. no use), mode of delivery, or infant sex (*P* > 0.05). Detailed estimates are given in Table 1.

### 3.2 Binary logistic regression analysis of factors associated with any direct breastfeeding during the first week after NICU discharge

Any DBF during the first 7 days after NICU discharge was the dependent variable (any DBF = 1, no DBF = 0). All variables examined in the univariate analysis were entered simultaneously, with study center retained as a fixed effect; continuous variables (anxiety score and paternal breastfeeding self-efficacy) were entered on their original scale, and categorical variables (oropharyngeal colostrum administration, severity of illness, DBF during hospitalization, educational level, monthly household income, and study center) were entered as dummy variables, as shown in Table 2. A full model containing all 28 variables was fitted first; variables with a Wald *P* < 0.05 in this full model were retained in a single step and refitted to give the final adjusted model, with study center retained regardless of its *P* value. The final model included 404 infants, of whom 213 had any DBF and 191 had no DBF. Collinearity diagnostics showed that the variance inflation factors for the continuous and dummy variables ranged from 1.051 to 1.838, all below 5, indicating no appreciable multicollinearity. The Hosmer-Lemeshow test gave a chi-squared of 7.316 (df = 8, *P* = 0.503), providing no evidence of a marked discrepancy between the predicted probabilities and the observed outcomes. In Box-Tidwell tests, the interaction terms for the anxiety score and the paternal breastfeeding self-efficacy score were not statistically significant (*P* = 0.817 and *P* = 0.650, respectively), providing no evidence that the relationship between these continuous predictors and the log odds of any DBF departed from linearity. After adjustment for the other variables in the final model, the adjusted odds of any DBF were higher for infants who received oropharyngeal colostrum at least once relative to those who never received it (aOR 5.582, 95% CI 2.477–12.581, *P* < 0.001), for infants who fed directly at the breast at least once during hospitalization relative to those who never did so (aOR 5.410, 95% CI 2.341–12.503, *P* < 0.001), and for mothers with a bachelor's degree or above relative to those who had completed junior college or below (aOR 4.776, 95% CI 1.970–11.581, *P* < 0.001). Each 1-point increase in the anxiety score was associated with lower adjusted odds of any DBF (aOR 0.902, 95% CI 0.851–0.957, *P* < 0.001), whereas each 1-point increase in the paternal breastfeeding self-efficacy score was associated with higher adjusted odds (aOR 1.069, 95% CI 1.044–1.095, *P* < 0.001). The overall association was statistically significant for severity of illness (chi-squared = 39.874, df = 3, *P* < 0.001), although the individual contrast of grade 2 versus grade 1 did not reach statistical significance (aOR 0.443, 95% CI 0.142–1.388, *P* = 0.162); the contrasts for grade 3 versus grade 1 (aOR 0.085, 95% CI 0.030–0.242, *P* < 0.001) and grade ≥4 versus grade 1 (aOR 0.018, 95% CI 0.005–0.074, *P* < 0.001) were both statistically significant. The overall association was also statistically significant for monthly household income (chi-squared = 9.674, df = 2, *P* = 0.008). Infants cared for at center 2 did not differ significantly from those at center 1 (aOR 1.833, 95% CI 0.825–4.073, *P* = 0.137). The complete results of the final model are given in Table 3.

**Table 2:** Coding of independent variables entered into the binary logistic regression analysis of factors associated with any direct breastfeeding during the first week after NICU discharge among preterm infants in the NICU.

Variable	Coding
Oropharyngeal colostrum administration	None = 0, ≥1 session = 1
Severity of illness	Dummy variables, with grade 1 as the reference (grade 2, grade 3, and grade ≥4)
DBF during hospitalization	None = 0, ≥1 session = 1
Educational level	Dummy variable, with junior college or below as the reference (bachelor's degree or above)
Monthly household income	Dummy variable, with <10,000 CNY as the reference (10,000–19,999 CNY, ≥20,000 CNY)
Study center	Dummy variable, with center 1 as the reference (center 2)
Anxiety score	Continuous variable
Paternal breastfeeding self-efficacy	Continuous variable

**Table 3:** Adjusted binary logistic regression analysis (aOR) of factors associated with any direct breastfeeding during the first week after NICU discharge among preterm infants in the NICU (*n* = 404; 213 with any DBF and 191 with no DBF).

Variable	*β*	SE	Wald	*P*	aOR (95% CI)	Overall *P*
Intercept	−1.818	1.110	2.682	0.101	0.162 (0.018, 1.430)	
Oropharyngeal colostrum administration						<0.001
None					1.000 (ref)	
≥1 session	1.720	0.415	17.200	<0.001	5.582 (2.477, 12.581)	
Severity of illness						<0.001
Grade 1					1.000 (ref)	
Grade 2	−0.813	0.582	1.952	0.162	0.443 (0.142, 1.388)	
Grade 3	−2.466	0.534	21.328	<0.001	0.085 (0.030, 0.242)	
Grade ≥4	−3.994	0.710	31.683	<0.001	0.018 (0.005, 0.074)	
DBF during hospitalization						<0.001
None					1.000 (ref)	
≥1 session	1.688	0.427	15.602	<0.001	5.410 (2.341, 12.503)	
Educational level						<0.001
Junior college or below					1.000 (ref)	
Bachelor's degree or above	1.564	0.452	11.975	<0.001	4.776 (1.970, 11.581)	
Monthly household income						0.008
<10,000 CNY					1.000 (ref)	
10,000-19,999 CNY	1.425	0.505	7.945	0.005	4.156 (1.543, 11.192)	
≥20,000 CNY	1.469	0.616	5.682	0.017	4.345 (1.298, 14.541)	
Study center						0.137
Center 1					1.000 (ref)	
Center 2	0.606	0.407	2.213	0.137	1.833 (0.825, 4.073)	
Anxiety score	-0.103	0.030	11.657	<0.001	0.902 (0.851, 0.957)	<0.001
Paternal breastfeeding self-efficacy score	0.067	0.012	29.586	<0.001	1.069 (1.044, 1.095)	<0.001

Coding of the dependent variable: any direct breastfeeding (exclusive or partial feeding at the breast) during the first 7 days after NICU discharge = 1, no DBF = 0. The model was fitted on the 404 infants with complete data for every variable in the model (213 with any DBF and 191 with no DBF); no imputation was performed. All estimates are adjusted odds ratios from the final multivariable model, and the reference category for each categorical variable is indicated in the table. Severity of illness was graded with the Neonatal Medical Index (NMI) and entered as four categories (grade 1, grade 2, grade 3, and grade ≥4; grades IV and V were combined because few infants were classified as grade V), with grade 1 as the reference category; none (no oropharyngeal colostrum administration), none (no DBF during hospitalization), junior college or below, <10,000 CNY, and center 1 were the reference categories for oropharyngeal colostrum administration, DBF during hospitalization, educational level, monthly household income, and study center, respectively. Study center was retained as a fixed effect. The odds ratio for each continuous predictor (anxiety score and paternal breastfeeding self-efficacy) represents the change in odds per one-unit increase. An overall *P* value is given for each multi-level categorical variable. Model fit: Hosmer-Lemeshow chi-squared = 7.316, df = 8, *P* = 0.503; variance inflation factors 1.051–1.838, all <5; Box-Tidwell tests for the anxiety score (*P* = 0.817) and the paternal breastfeeding self-efficacy score (*P* = 0.650) provided no evidence of departure from linearity. aOR, adjusted odds ratio; CI, confidence interval; NMI, Neonatal Medical Index.

## 4 Discussion

### 4.1 Status of direct breastfeeding in the first week after NICU discharge among preterm infants

Facilitating feeding at the breast should be a key goal of breastfeeding promotion programs in the NICU (9, 23), because it is associated with a higher volume of breast milk consumed and with longer breastfeeding duration (24). In practice, however, preterm infants admitted to the NICU depend largely on tube feeding or bottle feeding and therefore have little opportunity to feed at the breast, so feeding at the breast remains difficult to establish. In this study, 215 of the 421 preterm infants (51.07%) had any DBF during the first 7 days after NICU discharge, including 76 (18.05%) who fed exclusively at the breast and 139 (33.02%) who fed partly at the breast, while 206 (48.93%) had no DBF. Direct comparison with previous studies is limited by differences in gestational age distribution, NICU organization, the definition of DBF, and the time point of outcome assessment. NICUs in many other countries are fully open, allowing parents to enter the ward and take part in care so that infants can feed directly at the breast as soon as they are able to feed orally; this creates favorable conditions for DBF, and a study from the United States (14) reported that 51% of preterm infants were partly feeding at the breast at NICU discharge. Against this background, the proportion of infants feeding exclusively at the breast in the present cohort was low, and the observed proportion of any DBF (51.07%) is broadly comparable to, rather than clearly below, estimates reported elsewhere. Because the present outcome was ascertained over the first post-discharge week rather than at a single point in time, and because no accepted benchmark exists for this population, outcome definition, and measurement time point, we report the observed proportion without assigning a value judgment. Research on breastfeeding promotion in preterm infants should continue to focus on the barriers to feeding at the breast during the NICU stay and in the early post-discharge period.

### 4.2 Factors associated with any direct breastfeeding during the first week after NICU discharge

#### 4.2.1 Oropharyngeal colostrum administration

In the adjusted model, receipt of oropharyngeal colostrum administration was associated with higher odds of any DBF: infants who received colostrum at least once had 5.582 times the adjusted odds of any DBF compared with those who never received it (95% CI 2.477–12.581). This association should be interpreted cautiously and does not indicate that oropharyngeal colostrum administration itself causes subsequent DBF. Colostrum is rich in immunoglobulins, growth factors, and cytokines, and topical oropharyngeal application has been proposed to promote immune maturation of the oral mucosa and sensorimotor development in preterm infants. Studies have reported that oropharyngeal colostrum administration is associated with a lower incidence of late-onset sepsis, necrotizing enterocolitis, and feeding intolerance and with a shorter time to full enteral nutrition (25, 26), which may prepare infants for oral feeding (27). Maternal involvement in the procedure may also strengthen mothers' confidence in and motivation for breastfeeding, which could indirectly favor DBF. Dani et al. (13) likewise reported that oropharyngeal colostrum administration predicted DBF in very preterm infants. Importantly, the probability of receiving this intervention is likely to be associated with clinical stability, unit practices, parental involvement, and breastfeeding-support protocols, so the observed association may at least in part reflect residual confounding by these measured and unmeasured characteristics. Whether incorporating oropharyngeal colostrum administration into routine care would increase the proportion of infants feeding at the breast therefore remains a hypothesis requiring confirmation in interventional studies.

#### 4.2.2 Severity of illness

Greater severity of illness was associated with lower odds of any DBF. The overall association was statistically significant (*P* < 0.001), and the adjusted odds of any DBF fell progressively across categories relative to grade 1 illness: grade 2 (aOR 0.443, 95% CI 0.142–1.388), grade 3 (aOR 0.085, 95% CI 0.030–0.242), and grade ≥4 (aOR 0.018, 95% CI 0.005–0.074). The contrast for grade 2 versus grade 1 did not reach statistical significance on its own (*P* = 0.162), whereas those for grade 3 and grade ≥4 did (both *P* < 0.001). Compared with term infants, preterm infants have a poorer arousal response, less accurate tongue positioning, weaker sucking bursts, discoordination between tongue and jaw during sucking, and greater difficulty regulating breathing. Critically ill preterm infants often require respiratory support and an indwelling gastric tube, which may further weaken oral motor function and make the establishment of feeding at the breast more difficult. Prolonged use of endotracheal and gastric tubes also gives infants negative facial and oral experiences that may reduce their interest in oral feeding (28). These mechanisms are plausible interpretations rather than mechanisms demonstrated in the present data, because oral motor function was not directly measured. Clinicians should nevertheless consider creating opportunities for graded, step-by-step feeding at the breast in critically ill preterm infants as early as the infant's condition allows.

#### 4.2.3 DBF during hospitalization

Infants who fed directly at the breast at least once during hospitalization had 5.410 times the adjusted odds of any DBF during the first week after discharge compared with those who never did so (95% CI 2.341–12.503). This variable is conceptually very close to the primary outcome and may lie on the pathway linking earlier infant characteristics, hospital care, parental factors, and post-discharge feeding. It should therefore be regarded, at least in part, as a marker of previous breastfeeding success and as a possible intermediate step rather than as a confounder or as an independent cause of the outcome; consequently, its inclusion in the model may attenuate the estimated associations of upstream variables such as gestational age, severity of illness, and maternal characteristics. Practicing feeding at the breast during hospitalization may help preterm infants learn and consolidate sucking skills and may promote maternal milk production and the establishment of a feeding rhythm between mother and infant. Briere (29) reported that among mothers who breastfed directly at least once a day on average, 84% and 44% were still providing breast milk 1 and 4 months after NICU discharge, respectively, far higher than the corresponding proportions among mothers who breastfed directly less than once a day on average (23% and 6%). Pinchevski-Kadir (30) and colleagues similarly reported that direct feeding at the breast was associated with breastfeeding duration in preterm infants.

Initiating feeding directly at the breast during the NICU stay may therefore be important for achieving it later on. Before it is attempted, the infant should be assessed. Once the preterm infant has been weaned off mechanical ventilation, vital signs can be assessed serially; when the infant has no apnea, a body temperature of 36.5–37.5℃within 24 h, a stable resting heart rate, a transcutaneous oxygen saturation >88%, normal muscle tone and responses to stimulation, and is able to feed orally, the mother can be notified by telephone and invited to the NICU. There, under the guidance of a professional nurse, she may attempt feeding directly at the breast. The nurse guides the mother in holding the infant, positioning the breast, and assessing whether the infant latches effectively, monitors the infant's heart rate and transcutaneous oxygen saturation continuously throughout, and provides assistance as needed. This is carried out once daily until the infant makes a smooth transition to feeding directly at the breast. These care procedures describe the local practice of the participating units rather than interventions whose effectiveness was evaluated in this study.

#### 4.2.4 Maternal educational level and monthly household income

Higher maternal educational level and higher monthly household income were associated with higher odds of any DBF. Mothers with a bachelor's degree or above had 4.776 times the adjusted odds of any DBF compared with mothers who had completed junior college or below (95% CI 1.970–11.581), and families with a monthly income of 10,000–19,999 CNY and of ≥20,000 CNY had 4.156 times (95% CI 1.543–11.192) and 4.345 times (95% CI 1.298–14.541) the adjusted odds, respectively, compared with families with a monthly income below 10,000 CNY; the overall association with monthly household income was statistically significant (*P* = 0.008). Mothers with higher educational and economic status tend to have better health literacy, more sources of breastfeeding information, and a stronger preference for breastfeeding as the optimal feeding method (15, 16). Breastfeeding education and support should therefore be prioritized for mothers with lower educational levels and lower household incomes.

#### 4.2.5 Maternal anxiety

Each 1-point increase in the anxiety score was associated with approximately 10% lower adjusted odds of any DBF (aOR 0.902, 95% CI 0.851–0.957). During the NICU stay, mothers face multiple stressors, including separation from their infant and uncertainty about the infant's condition; elevated anxiety may blunt the sensitivity and confidence of maternal feeding interactions and thereby hinder the establishment of feeding at the breast. Studies in China have reported a high prevalence of anxiety and depression among mothers of preterm infants in the NICU, associated with delayed onset of lactation and prolonged mother-infant separation (31). An important caveat is that the SAS was administered shortly before discharge, by which time mothers had already accumulated experience of success or difficulty with feeding at the breast. The observed association may therefore be bidirectional: higher anxiety may reduce the likelihood of feeding at the breast, but difficulty with feeding may equally increase maternal anxiety. Because both variables were measured at the same time point, the direction of the association cannot be determined from these data, and this association should not be interpreted as a causal effect. Screening for anxiety and providing psychological support to mothers of preterm infants should nevertheless be considered an integral part of NICU care.

#### 4.2.6 Paternal breastfeeding self-efficacy

Breastfeeding self-efficacy refers to a mother's confidence in her ability to carry out breastfeeding behaviors. It was paternal, rather than maternal, breastfeeding self-efficacy that was retained in the final adjusted model: each 1-point increase in paternal breastfeeding self-efficacy was associated with 6.9% higher adjusted odds of any DBF, and expressed on a clinically more interpretable scale, a 10-point increase corresponds to an aOR of approximately 1.95 (95% CI 1.54–2.48). Maternal breastfeeding self-efficacy differed between the two feeding groups in the univariate analysis but was not retained once paternal self-efficacy and the other variables were taken into account. In the full model, maternal breastfeeding self-efficacy had an aOR of 1.023 (95% CI 0.964–1.087, *P* = 0.451) and paternal breastfeeding self-efficacy had an aOR of 1.053 (95% CI 1.006–1.102, *P* = 0.026), so the estimates were similar in the full and final models and maternal self-efficacy was not statistically significant in either. This pattern reflects the variable-selection procedure rather than evidence that paternal self-efficacy is causally more important than maternal self-efficacy; the two measures may be correlated, and the difference should be interpreted cautiously. Fathers are a principal source of instrumental and emotional support for mothers in the postpartum period, and fathers with higher breastfeeding self-efficacy may be more likely to take on practical tasks, to protect the mother's time for feeding or expressing, and to encourage continued feeding when difficulties arise, all of which may increase the opportunity for feeding directly at the breast (19, 21). Parents with higher self-efficacy may be more likely to persist with breastfeeding and to seek solutions when difficulties arise rather than give up. As with anxiety, however, the temporal relationship is not established: the FBSES-SF was completed shortly before discharge, so previous experience of successful feeding may itself have raised the father's confidence. The association should therefore be interpreted as bidirectional rather than causal. Mills et al. (14) reported that breastfeeding self-efficacy is an important predictor of feeding at the breast in preterm infants, and Brockway et al. (32) found that mothers with higher breastfeeding self-efficacy on admission were more likely to have infants who were exclusively breastfed at discharge; the present findings suggest that the father's self-efficacy deserves similar attention.

Taken together, this multivariable analysis identified factors associated with any DBF during the early post-discharge period at three levels: the infant, feeding practice, and parental support. At the infant level, greater severity of illness reflected a heavier disease burden and objectively constrained opportunities for feeding at the breast, and it showed a graded association with the outcome that was statistically significant overall. Study center was retained as a fixed effect but was not independently associated with the outcome. At the level of feeding practice, oropharyngeal colostrum administration and feeding at the breast during hospitalization were the variables most strongly associated with the outcome, although both may partly reflect previous feeding success or unit practice rather than independent effects. At the parental level, maternal educational level, monthly household income, maternal anxiety, and paternal breastfeeding self-efficacy were associated with the outcome, with the latter two potentially bidirectional; because it was the father's self-efficacy that was retained in the adjusted model, interventions that involve fathers in breastfeeding support may be particularly relevant. Most of these variables represent potential targets for future interventional evaluation through standardized protocols rather than established intervention components, and the high-risk groups identified here - mothers with lower educational attainment, lower household income, and higher levels of anxiety, and infants of greater illness severity - warrant early identification and targeted support. Health education, psychological support, and practical skills coaching may help to strengthen breastfeeding self-efficacy and the family's capacity to support feeding. A systematic approach involving early assessment, timely support, and sustained follow-up throughout the hospital stay and the early post-discharge period may help maintain breastfeeding after discharge; whether it increases the proportion of infants feeding at the breast has not been demonstrated and should be tested prospectively.

### 4.3 Limitations

This study has several limitations. First, convenience sampling was used and only two NICUs participated, so selection bias cannot be excluded and the findings may not be generalizable to other hospitals, regions, or NICU organizational models. Second, although study center was retained as a fixed effect in the adjusted model, possible residual center effects - differences between the two hospitals in clinical severity, parental access, breastfeeding support, kangaroo mother care, family-centered care, feeding protocols, and discharge practices - could have influenced both exposures and the outcome, and residual confounding by unmeasured hospital- and family-level factors cannot be ruled out. Third, a large number of variables were examined relative to the number of outcome events, and the final model was obtained by retaining from a full multivariable model only those variables with a Wald *P* < 0.05; this data-driven strategy may have omitted important confounders, may be sensitive to the particular sample analyzed, and may carry a risk of overfitting and model instability. The variables retained should therefore not be interpreted as representing all independent determinants of the outcome. Fourth, exclusive and partial feeding at the breast were combined into a single binary outcome, which simplified the analysis but necessarily discarded information and obscured clinically meaningful differences between these feeding patterns. Fifth, some feeding information was based on parental recall, so recall bias may have occurred, and the accuracy of parental report was not validated against an independent feeding diary in all cases. Sixth, anxiety and depression were self-reported using the SAS and EPDS, respectively, and may have been subject to social desirability bias. Seventh, maternal anxiety and paternal breastfeeding self-efficacy were measured shortly before discharge, close in time to the outcome, so reverse causation cannot be excluded. Eighth, the observational design cannot establish that the identified associations are causal or that the associated factors are modifiable; in particular, although DBF during hospitalization may act as a mediator on the pathway to post-discharge feeding, this possibility could not be resolved, no formal mediation analysis was performed. A sensitivity analysis excluding DBF during hospitalization, however, left all retained associations essentially unchanged (Supplementary Table 6), indicating that they were not materially mediated by DBF during hospitalization. Ninth, the study did not include all potentially relevant influences, such as the attitudes of healthcare professionals and hospital policy support. Tenth, denominators differed between variables in Table 1 because a small number of clinical variables had missing values; no imputation was performed and each analysis used all available data, which may have reduced precision for the affected variables. Missing values were more concentrated in the no-DBF group (Supplementary Table 5), so the complete-case analysis could have introduced some selection bias if the missingness was not completely at random. Future research should adopt multicenter, large-sample designs with longitudinal follow-up, employ a prespecified adjustment set based on clinical and epidemiological reasoning, and examine combined interventions addressing oropharyngeal colostrum administration, feeding at the breast during hospitalization, and parental psychological state and breastfeeding self-efficacy, in order to generate more representative conclusions.

## 5 Conclusion

In this cohort of 421 preterm infants from two Chinese NICUs, the proportion of infants who fed directly at the breast at least once during the first 7 days after NICU discharge was 51.07%, and 18.05% fed directly exclusively at the breast. In the adjusted analysis (*n* = 404), receipt of oropharyngeal colostrum administration, feeding at the breast during hospitalization, higher maternal educational level, higher monthly household income, and higher paternal breastfeeding self-efficacy were associated with higher odds of any DBF, whereas greater severity of illness and higher maternal anxiety score were associated with lower odds. Because the study was observational and several measurements were concurrent with the outcome, these findings describe associations rather than causal effects, and the associated factors identified here - particularly oropharyngeal colostrum administration, feeding at the breast during hospitalization, maternal anxiety, and paternal breastfeeding self-efficacy - represent potential targets for future interventional evaluation rather than established intervention components. The results support early assessment of feeding readiness and targeted support for mothers with lower educational attainment, lower household income, or higher anxiety, with the aim of facilitating feeding at the breast during the early post-discharge period.

## Data Availability

The original contributions presented in the study are included in the article/Supplementary Material, further inquiries can be directed to the corresponding author.
