# Supplementary material for: Direct breastfeeding during the first week after NICU discharge among preterm infants: prevalence and associated factors
Source: Front Pediatr. 2026 Oct 30;14:2002068. doi: 10.3389/fped.2026.2002068 (PMC13759478; doi:10.3389/fped.2026.2002068)
Supplement: Supplementary file 1 [file Table1.docx]

**STROBE Statement—checklist of items that should be included in reports of cohort studies**

**Manuscript:** “Direct breastfeeding during the first week after NICU discharge among preterm infants: prevalence and associated factors” (Frontiers in Pediatrics, Original Research).

Version used: the study is a two-center prospective cohort of preterm infant–parent units recruited consecutively during NICU hospitalization, with exposure and covariate measurements made before discharge and the outcome ascertained over a defined follow-up interval (feeding status during the first 7 days after NICU discharge); follow-up was complete for all enrolled units. The cohort version of the STROBE checklist is therefore the appropriate one, and it corresponds to the design implemented.

| **Item No.** | **Recommendation** | **Reported in the revised manuscript** |
| --- | --- | --- |
| **Title and abstract** | | |
| 1 | (a) Indicate the study’s design with a commonly used term in the title or the abstract. (b) Provide in the abstract an informative and balanced summary of what was done and what was found. | Abstract, p. 1-3, lines 19-60. The design is named in the Abstract under Methods (“a multicenter prospective cohort study”); the title is descriptive (Title, p. 1, line 1). The structured abstract (Background/Methods/Results/Conclusions) reports the setting, sampling, instruments, the numbers enrolled and analysed (421; 404 in the adjusted model), the prevalence estimates, the adjusted odds ratios with 95% confidence intervals, and the associational (non-causal) interpretation. |
| **Introduction** | | |
| 2 | Background/rationale: explain the scientific background and rationale for the investigation being reported. | Introduction, p. 3-5, lines 61-109. Covers the burden in preterm infants, the distinction between feeding at the breast and receiving expressed human milk, reported rates of direct breastfeeding (DBF) of 10%–52.4%, the WHO/World Health Assembly breastfeeding target, and the limited Chinese evidence on DBF in the early post-discharge period. |
| 3 | Objectives: state specific objectives, including any prespecified hypotheses. | Introduction, p. 3-5, lines 61-109, final paragraph: “This study therefore aimed to describe the proportion of preterm infants feeding directly at the breast during the first 7 days after NICU discharge and to examine factors associated with this outcome, in order to inform future intervention development.” No formal a priori hypothesis was stated; the analysis was exploratory. |
| **Methods** | | |
| 4 | Study design: present key elements of study design early in the paper. | Methods 2.1, p. 5-7, lines 111-156. Multicenter prospective observational cohort study in the NICUs of two tertiary hospitals; consecutive convenience sampling; the six-step temporal sequence is spelled out (screening and enrolment, clinical data collection, questionnaire administration, discharge, 1-week post-discharge assessment, completion of the analytic dataset). |
| 5 | Setting: describe the setting, locations, and relevant dates, including periods of recruitment, exposure, follow-up, and data collection. | Methods 2.1, p. 5-7, lines 111-156. Shanghai First Maternity and Infant Hospital (Shanghai) and Taicang First People’s Hospital (Taicang, Jiangsu Province); recruitment July 2025 to July 2026. The recruitment window is repeated in the Ethics statement, p. 31-32, lines 689-703. |
| 6 | (a) Give the eligibility criteria, and the sources and methods of selection of participants. Describe methods of follow-up.  (b) For matched studies, give matching criteria and number of exposed and unexposed. | Methods 2.1, p. 5-7, lines 111-156. Inclusion and exclusion criteria for infants and for parents; method of selection = all eligible families admitted during the recruitment period approached consecutively; no additional selection beyond the eligibility criteria. Participant flow is given in the same section and in Figure 1 (provided as a separate figure file). Methods of follow-up: the cohort was followed prospectively from NICU admission to the 1-week post-discharge assessment; feeding status was ascertained at a single post-discharge follow-up (outpatient visit or telephone interview) covering the whole first post-discharge week, and all 421 enrolled infant–parent units (100%) completed this follow-up. No matching was performed, as this was an unmatched consecutive cohort. |
| 7 | Variables: clearly define all outcomes, exposures, predictors, potential confounders, and effect modifiers. Give diagnostic criteria, if applicable. | Outcome: Methods 2.3, p. 9-12, lines 217-280 — three mutually exclusive categories (exclusive, partial, no DBF) plus a derived binary outcome (any DBF), with the rationale for combining exclusive and partial DBF into the binary outcome and the reason for labelling the reference category “no DBF” rather than “bottle feeding”. Predictors and covariates: Methods 2.2.1, p. 7-8, lines 158-183 — the general information questionnaire, with the operational definition of every hospitalisation variable, the 0.2–0.5 mL/session definition of oropharyngeal colostrum administration, the definition of a DBF session, and the Neonatal Medical Index grading of severity of illness including the reason why grades IV and V were combined into a single category; Methods 2.3, p. 9-12, lines 217-280 gives the timing of every measurement. Variable coding in the model: Table 2, p. 20, lines 424-426. No variable was pre-specified as an a priori confounder (Methods 2.4, p. 12-15, lines 281-352). |
| 8* | Data sources/measurement: for each variable of interest, give sources of data and details of methods of assessment (measurement). Describe comparability of assessment methods if there is more than one group. | Methods 2.2.1-2.2.5, p. 7-9, lines 158-216. General information questionnaire; BSES-SF; FBSES-SF; EPDS; SAS — for each: original source, Chinese validation, number of items, scoring, cut-offs, and Cronbach’s alpha. The same instruments and the same administration procedure were used at both centers. Methods 2.3, p. 9-12, lines 217-280. Three data-collection time points; infant clinical variables abstracted from the medical record; hospitalisation practices recorded on the ward; questionnaires completed independently and checked on site; feeding status reported by the mother at the 1-week follow-up and cross-checked against the parents’ feeding record and, where the infant attended, the clinical record. |
| 9 | Bias: describe any efforts to address potential sources of bias. | Methods 2.3, p. 9-12, lines 217-280. Standardised instructions from uniformly trained investigators; mothers and fathers completed questionnaires independently; questionnaires checked on site and incomplete or contradictory items clarified with the respondent; items were never revised on the investigator’s own judgment; the investigator was unaware of the post-discharge outcome when checking the questionnaires; feeding status cross-checked against a second source. Methods 2.4, p. 12-15, lines 281-352. Complete-case analysis with explicit diagnostic checks (variance inflation factor, Hosmer–Lemeshow, Box–Tidwell). Limitations 4.3, p. 29-31, lines 631-671 discusses the biases that could not be eliminated (selection bias, residual confounding, overfitting, recall bias, social desirability bias, reverse causation, missing data), with the likely direction and magnitude of their effect. |
| 10 | Study size: explain how the study size was arrived at. | Methods 2.4, p. 12-15, lines 281-352, “Sample-size justification”. Sample size was based on the number of outcome events rather than on the total number of participants, with an anticipated proportion of any DBF of approximately 50%. 27 variables initially considered = 31 slope parameters; 23 candidates = 27 parameters; 404 infants had complete data (213 with any DBF, 191 with no DBF); the final model contained 11 slope parameters plus one intercept, i.e. 19.36 events and 17.36 non-events per parameter. The earlier target of 297–446 participants was derived from 10–15 participants per variable with a further 10% allowance for invalid questionnaires. |
| 11 | Quantitative variables: explain how quantitative variables were handled in the analyses. If applicable, describe which groupings were chosen and why. | Methods 2.4, p. 12-15, lines 281-352. Continuous variables were entered on their original scale and were not categorised (anxiety score and paternal breastfeeding self-efficacy; confirmed in Table 2, p. 20, lines 424-426). Categorical variables were entered as dummy variables. Groupings used: severity of illness (Neonatal Medical Index grade 1, grade 2, grade 3, and grade ≥4), educational level (junior college or below; bachelor’s degree or above), monthly household income (<10,000; 10,000–19,999; ≥20,000 CNY), oropharyngeal colostrum administration and DBF during hospitalization (none; ≥1 session), and study center (center 1; center 2) — Table 2, p. 20, lines 424-426 and Table 3 note, p. 21-22, lines 428-450. Groupings chosen and why: the five Neonatal Medical Index grades were collapsed into four categories (grade 1, grade 2, grade 3, and grade ≥4) by combining grades IV and V, because few infants were classified as grade V; this is stated in Methods 2.2.1, p. 7-8, lines 158-183. Continuous variables were not grouped. Category boundaries for all other categorical variables are given in Table 1, p. 16-19, with the denominator of each variable in the Table 1 note, p. 16-19, lines 372-385. |
| 12 | (a) Describe all statistical methods, including those used to control for confounding. (b) Describe any methods used to examine subgroups and interactions. (c) Explain how missing data were addressed. (d) If applicable, describe analytical methods taking account of sampling strategy. (e) Describe any sensitivity analyses. | (a) Methods 2.4, p. 12-15, lines 281-352 (descriptive statistics and tests; the multivariable strategy; model diagnostics), Results 3.2, p. 19-20, lines 386-423, and Table 2, p. 20, lines 424-426. Confounding was addressed by entering all variables examined in the univariate analysis simultaneously, together with study center retained as a fixed effect, into a full model and then retaining, in a single step, the variables with a Wald P < 0.05 and refitting them; the complete results of the full model are given in Supplementary Table 3 (separate Supplementary Material file), p. 3-4. (b) No subgroup or interaction analyses were pre-specified or performed. The only product terms fitted were the Box–Tidwell diagnostic terms used to test the linearity of the two continuous predictors (Methods 2.4, p. 12-15, lines 281-352; Results 3.2, p. 19-20, lines 386-423). (c) Methods 2.3, p. 9-12, lines 217-280 gives the number and percentage missing for each variable (the scores of the four scales were complete for all participants; no imputation was performed and all available data were used), and Methods 2.4, p. 12-15, lines 281-352 states that 404 of 421 infant-parent units entered the complete-case regression analysis. The consequences are discussed in Limitations 4.3, p. 29-31, lines 631-671, item Tenth. (d) Consecutive convenience sampling of all eligible families was used, without sampling weights or a complex survey design; accordingly no design-based analysis was applied. Stated in Methods 2.1, p. 5-7, lines 111-156 and acknowledged in Limitations 4.3, p. 29-31, lines 631-671, item First. (e) A sensitivity analysis excluding DBF during hospitalization was performed and left all retained associations essentially unchanged (Supplementary Table 6). The manuscript states explicitly that the analysis was associational rather than causal and that no formal mediation analysis was carried out (Methods 2.4, p. 12-15, lines 281-352, “Conceptual role of DBF during hospitalization”; Limitations 4.3, p. 29-31, lines 631-671, item Eighth). |
| **Results** | | |
| 13* | (a) Report numbers of individuals at each stage of study—e.g. numbers potentially eligible, examined for eligibility, confirmed eligible, included in the study, completing follow-up, and analysed. (b) Give reasons for non-participation at each stage. (c) Consider use of a flow diagram. | (a) Methods 2.1, p. 5-7, lines 111-156 and Figure 1 (provided as a separate figure file): 446 preterm infants were screened and assessed for eligibility → 10 excluded → 436 eligible families approached → 15 declined → 421 enrolled → 421 completed the discharge assessment and the 1-week post-discharge follow-up. Univariate analysis: 421; multivariable analysis: 404 (213 with any DBF and 191 with no DBF). (b) Methods 2.1, p. 5-7, lines 111-156: exclusions (severe congenital disease requiring surgery, n = 3; necrotizing enterocolitis, n = 3; breast milk jaundice, n = 4) and non-participation (15 families declined, being unwilling to complete the questionnaire or the 1-week follow-up). (c) Figure 1 (provided as a separate figure file). |
| 14* | (a) Give characteristics of study participants and information on exposures and potential confounders. (b) Indicate the number of participants with missing data for each variable of interest.  (c) Summarise follow-up time (e.g. average and total amount). | (a) Results 3.1, p. 15-19, lines 354-385 and Table 1, p. 16-19, with its title and note: demographic, clinical, and social characteristics by feeding group, given as median (IQR) or mean ± SD and as frequency (%). (b) Methods 2.3, p. 9-12, lines 217-280 reports the missing values per variable (ventricular septal defect or patent ductus arteriosus, 14 infants, 3.33%; duration of CPAP and frequency of breastfeeding or pumping within 48 h after delivery, 2 infants each, 0.48%; each remaining variable, 1 infant, 0.24%; the four scale scores were complete for all 421 participants). The denominator for every variable is given in the Table 1 note, p. 16-19, lines 372-385, and Supplementary Table 5 (separate Supplementary Material file), p. 6-7 reports the number of missing values for each variable, overall and by feeding group; n = 404 with complete data in the adjusted model (Table 3 title, p. 21, lines 428-430).  (c) Follow-up was a single fixed interval: the outcome was ascertained once, at the 1-week post-discharge follow-up, and covered the whole first week after discharge. All 421 enrolled infant–parent units (100%) completed this follow-up, so there was no loss to follow-up; the total follow-up time was 421 infant-weeks (7 days per infant). |
| 15* | Outcome data: report numbers of outcome events or summary measures over time. | Results 3.1, p. 15-19, lines 354-385 and Table 1, p. 16-19. Any DBF 215/421 (51.07%); exclusive DBF 76 (18.05%); partial DBF 139 (33.02%); no DBF 206 (48.93%). The outcome was ascertained over the whole first week after discharge rather than at a single point in time. In the adjusted model: 213 of 404 (52.7%) with any DBF and 191 (47.3%) with no DBF (Results 3.2, p. 19-20, lines 386-423; Table 3 title, p. 21, lines 428-430). |
| 16 | (a) Give unadjusted estimates and, if applicable, confounder-adjusted estimates and their precision. Make clear which confounders were adjusted for and why they were included. (b) Report category boundaries when continuous variables were categorized. (c) If relevant, consider translating estimates of relative risk into absolute risk for a meaningful time period. | (a) Unadjusted estimates: Table 1, p. 16-19 and Results 3.1, p. 15-19, lines 354-385. Adjusted estimates with 95% confidence intervals: Results 3.2, p. 19-20, lines 386-423, Table 3 title, p. 21, lines 428-430 and Table 3 note, p. 21-22, lines 428-450, and the Abstract, p. 1-3, lines 19-60. The variables entered into the adjusted model, and the reason for including each, are documented in Methods 2.4, p. 12-15, lines 281-352, “Variable selection”, and in Supplementary Table 2 (separate Supplementary Material file), p. 1-2, which lists the 27 variables considered a priori, the 23 eligible, and the four excluded with the reason for each exclusion; no variable was pre-specified as an a priori confounder, and study center was retained as a fixed effect. The complete results of the full model are given in Supplementary Table 3 (separate Supplementary Material file), p. 3-4. (b) Continuous predictors were entered on their original scale and were not categorised (Table 2, p. 20, lines 424-426). The category boundaries of the categorical variables analysed are given in Table 2, p. 20, lines 424-426 and the Table 3 note, p. 21-22, lines 428-450, and, for all remaining variables, in Table 1, p. 16-19. The overall P value is reported for each multi-level categorical variable. (c) Not applicable: the outcome is a binary feeding status rather than a time-to-event outcome, and no absolute risk or risk difference was computed. |
| 17 | Other analyses: report other analyses done — e.g. analyses of subgroups and interactions, and sensitivity analyses. | Model diagnostics are reported in Results 3.2, p. 19-20, lines 386-423 (variance inflation factors 1.051–1.838; Hosmer–Lemeshow χ² = 7.316, df = 8, P = 0.503; Box–Tidwell P = 0.817 and 0.650), with the corresponding methods in Methods 2.4, p. 12-15, lines 281-352. No subgroup or interaction analyses were performed; a sensitivity analysis excluding DBF during hospitalization left all retained associations essentially unchanged (Supplementary Table 6). Supplementary Table 2 (separate Supplementary Material file), p. 1-2 reports the fate of all variables considered for the model, Supplementary Table 3 (separate Supplementary Material file), p. 3-4 the complete results of the full multivariable model, and Supplementary Table 4 (separate Supplementary Material file), p. 5 the comparison of baseline and care-practice characteristics between the two centers. |
| **Discussion** | | |
| 18 | Key results: summarise key results with reference to study objectives. | Discussion 4.1, p. 22-23, lines 452-475; the summary paragraph of Discussion 4.2, p. 23-29, lines 476-630; Conclusion 5, p. 31, lines 672-688. |
| 19 | Limitations: discuss limitations of the study, taking into account sources of potential bias or imprecision. Discuss both direction and magnitude of any potential bias. | Limitations 4.3, p. 29-31, lines 631-671. Ten numbered limitations: convenience sampling and limited generalisability (First); residual center effects and unmeasured confounding (Second); data-driven variable selection with a risk of overfitting (Third); the combination of exclusive and partial DBF into a single binary outcome (Fourth); recall bias from parental report of feeding (Fifth); social desirability bias in the self-reported psychological measures (Sixth); reverse causation, because several exposures were measured close in time to the outcome (Seventh); the absence of causal inference and of formal mediation analysis (Eighth); omission of other potentially relevant influences such as health-professional attitudes and hospital policy (Ninth); and differing denominators caused by missing data, which were more concentrated in the no-DBF group (Tenth). |
| 20 | Interpretation: give a cautious overall interpretation of results considering objectives, limitations, multiplicity of analyses, results from similar studies, and other relevant evidence. | Discussion 4.2.1-4.2.6, p. 23-29, lines 478-630; the summary paragraph of Discussion 4.2, p. 23-29, lines 476-630; Conclusion 5, p. 31, lines 672-688. Each subsection compares the present findings with the existing literature and states explicitly that the associations are not causal and that several exposures were measured concurrently with the outcome. |
| 21 | Generalisability: discuss the generalisability (external validity) of the study results. | Limitations 4.3, p. 29-31, lines 631-671, item First and the closing paragraph: convenience sampling and only two participating NICUs limit generalisability to other hospitals, regions, and NICU organisational models; a multicenter, large-sample design with longitudinal follow-up is recommended for future work. |
| **Other information** | | |
| 22 | Funding: give the source of funding and the role of the funders for the present study. | Funding, p. 33, lines 718-720. No financial support was received for this study. |

From: von Elm E, Altman DG, Egger M, Pocock SJ, Gøtzsche PC, Vandenbroucke JP; STROBE Initiative. The Strengthening the Reporting of Observational Studies in Epidemiology (STROBE) statement: guidelines for reporting observational studies. Lancet. 2007;370(9596):1453-7. doi: 10.1016/S0140-6736(07)61602-X.
