# Supplementary material for: Direct breastfeeding during the first week after NICU discharge among preterm infants: prevalence and associated factors
Source: Front Pediatr. 2026 Oct 30;14:2002068. doi: 10.3389/fped.2026.2002068 (PMC13759478; doi:10.3389/fped.2026.2002068)
Supplement: Supplementary file 2 [file Table2.docx]

**Supplementary Material**

Supplementary Table 1. Grading criteria of the Neonatal Medical Index (NMI) used to classify severity of illness.

| **Grade** | **Criteria** |
| --- | --- |
| I | Birth weight > 1,000 g; no respiratory distress and no other serious complications. |
| II | Birth weight > 1,500 g; assisted ventilation ≤ 48 h; no periventricular or intraventricular hemorrhage. |
| III | Assisted ventilation 3-14 days or birth weight < 1,000 g; periventricular-intraventricular hemorrhage grade 1 or 2, etc. |
| IV | Assisted ventilation 15-28 days; respiratory distress or bradycardia requiring resuscitation, etc. |
| V | Assisted ventilation ≥ 29 days; confirmed or suspected meningitis; seizures; periventricular-intraventricular hemorrhage grade 3 or 4, etc. |

Note: The NMI classifies preterm infants from grade I (least severe) to grade V (most severe). It has shown good predictive and temporal validity and can distinguish feeding status across levels of illness severity in preterm infants. Grading is hierarchical: criteria are evaluated from grade V down to grade I, and each infant is assigned to the highest grade whose criteria are met; when criteria for more than one grade are present concurrently, the more severe grade is assigned. Adapted from Korner et al. (17); the Chinese version and its use in the NICU preterm population follow Sun et al. (18).

Supplementary Table 2. Independent variables initially considered for the multivariable logistic regression, their eligibility for the original multivariable model, and the reason for any exclusion.

| **Variable** | **Type** | **Status in the original multivariable analysis** |
| --- | --- | --- |
| Total length of hospital stay (days) | Continuous | Eligible |
| Duration of mechanical ventilation (days) | Continuous | Excluded: did not differ between the feeding groups in the univariate comparison (P = 0.731) |
| Duration of CPAP (days) | Continuous | Eligible |
| Gestational age at first oral feeding (weeks) | Continuous | Eligible |
| Time to regain birth weight (days) | Continuous | Eligible |
| Days with an indwelling gastric tube (days) | Continuous | Eligible |
| Mode of delivery | Categorical | Excluded: did not differ between the feeding groups in the univariate comparison (P = 0.071) |
| Ventricular septal defect or patent ductus arteriosus | Categorical | Eligible |
| Oropharyngeal colostrum administration | Categorical | Eligible |
| Infant sex | Categorical | Excluded: did not differ between the feeding groups in the univariate comparison (P = 0.307) |
| Birth weight (g) | Continuous | Eligible |
| Severity of illness (NMI grade) | Categorical | Eligible |
| Gestational age at birth (weeks) | Continuous | Eligible |
| Admission to a family-centred ward | Categorical | Eligible |
| DBF during hospitalization | Categorical | Eligible |
| Kangaroo mother care | Categorical | Eligible |
| Type of medical insurance | Categorical | Eligible |
| Educational level | Categorical | Eligible |
| Monthly household income | Categorical | Eligible |
| Time to initiate milk expression after delivery | Categorical | Eligible |
| Breast pump use within 6 h after delivery | Categorical | Excluded: inadvertently omitted from the original analysis; reinstated in the revised model |
| Frequency of breastfeeding or pumping within 48 h after delivery (times) | Continuous | Eligible |
| Breast massage within 24 h after delivery | Categorical | Eligible |
| Depression score (points) | Continuous | Eligible |
| Anxiety score (points) | Continuous | Eligible |
| Maternal breastfeeding self-efficacy (points) | Continuous | Eligible |
| Paternal breastfeeding self-efficacy (points) | Continuous | Eligible |

Note: Twenty-seven variables were considered a priori on the basis of the literature and clinical reasoning. Four were not eligible for the original multivariable model: three (duration of mechanical ventilation, mode of delivery, and infant sex) because they did not differ between the feeding groups in the univariate comparison (P >0.05), and one (breast pump use within 6 h after delivery) because it had been inadvertently omitted. In the revised analysis, duration of mechanical ventilation was re-expressed as mechanical ventilation (use vs. no use), breast pump use within 6 h after delivery was reinstated, and study center was added, so that the full multivariable model contained 28 variables. NMI, Neonatal Medical Index; DBF, direct breastfeeding; CPAP, continuous positive airway pressure.

**Supplementary Table 3. Results of the full multivariable logistic regression model, in which all 28 variables were entered simultaneously (n = 404).**

| **Variable** | **β** | **SE** | **Wald** | **P** | **aOR (95% CI)** | **Overall P** |
| --- | --- | --- | --- | --- | --- | --- |
| Intercept | -20.502 | 10.165 | 4.068 | 0.044 | 0.000 (0.000, 0.561) |  |
| Mode of delivery |  |  |  |  |  | 0.545 |
| Vaginal delivery |  |  |  |  | 1.000 (ref) |  |
| Cesarean section | -0.399 | 0.660 | 0.366 | 0.545 | 0.671 (0.184, 2.446) |  |
| Ventricular septal defect or patent ductus arteriosus |  |  |  |  |  | 0.306 |
| Yes |  |  |  |  | 1.000 (ref) |  |
| No | 0.548 | 0.535 | 1.048 | 0.306 | 1.730 (0.606, 4.938) |  |
| Oropharyngeal colostrum administration |  |  |  |  |  | <0.001 |
| None |  |  |  |  | 1.000 (ref) |  |
| ≥1 session | 1.935 | 0.529 | 13.369 | <0.001 | 6.927 (2.455, 19.550) |  |
| Sex |  |  |  |  |  | 0.873 |
| Male |  |  |  |  | 1.000 (ref) |  |
| Female | 0.076 | 0.478 | 0.025 | 0.873 | 1.079 (0.423, 2.755) |  |
| Severity of illness |  |  |  |  |  | 0.038 |
| Grade 1 |  |  |  |  | 1.000 (ref) |  |
| Grade 2 | -0.153 | 0.713 | 0.046 | 0.830 | 0.858 (0.212, 3.471) |  |
| Grade 3 | -1.669 | 0.696 | 5.754 | 0.016 | 0.188 (0.048, 0.737) |  |
| Grade ≥4 | -2.307 | 1.057 | 4.764 | 0.029 | 0.100 (0.013, 0.790) |  |
| Admission to family-centered ward |  |  |  |  |  | 0.245 |
| Yes |  |  |  |  | 1.000 (ref) |  |
| No | 0.641 | 0.551 | 1.354 | 0.245 | 1.898 (0.645, 5.589) |  |
| DBF during hospitalization |  |  |  |  |  | 0.001 |
| None |  |  |  |  | 1.000 (ref) |  |
| ≥1 session | 1.791 | 0.548 | 10.688 | 0.001 | 5.994 (2.049, 17.538) |  |
| Kangaroo mother care |  |  |  |  |  | 0.201 |
| None |  |  |  |  | 1.000 (ref) |  |
| ≥1 session | 0.700 | 0.547 | 1.635 | 0.201 | 2.013 (0.689, 5.883) |  |
| Type of medical insurance |  |  |  |  |  | 0.372 |
| Urban resident basic medical insurance |  |  |  |  | 1.000 (ref) |  |
| Urban employee basic medical insurance | 0.464 | 0.519 | 0.798 | 0.372 | 1.590 (0.575, 4.397) |  |
| Educational level |  |  |  |  |  | 0.047 |
| Junior college or below |  |  |  |  | 1.000 (ref) |  |
| Bachelor's degree or above | 1.285 | 0.648 | 3.933 | 0.047 | 3.613 (1.015, 12.861) |  |
| Monthly household income (CNY) |  |  |  |  |  | 0.012 |
| <10,000 |  |  |  |  | 1.000 (ref) |  |
| 10,000–19,999 | 1.698 | 0.602 | 7.967 | 0.005 | 5.462 (1.680, 17.758) |  |
| ≥20,000 | 1.539 | 0.820 | 3.518 | 0.061 | 4.658 (0.933, 23.246) |  |
| Time to initiate milk expression after delivery |  |  |  |  |  | 0.533 |
| Within 6 h |  |  |  |  | 1.000 (ref) |  |
| Within 12 h | 0.366 | 0.743 | 0.243 | 0.622 | 1.442 (0.336, 6.185) |  |
| >12 h | -0.503 | 0.880 | 0.326 | 0.568 | 0.605 (0.108, 3.396) |  |
| Breast pump use within 6 h after delivery |  |  |  |  |  | 0.794 |
| Yes |  |  |  |  | 1.000 (ref) |  |
| No | -0.156 | 0.599 | 0.068 | 0.794 | 0.856 (0.265, 2.767) |  |
| Breast massage within 24 h after delivery |  |  |  |  |  | 0.483 |
| Yes |  |  |  |  | 1.000 (ref) |  |
| No | 0.433 | 0.618 | 0.492 | 0.483 | 1.542 (0.460, 5.174) |  |
| Study center |  |  |  |  |  | 0.062 |
| Center 1 |  |  |  |  | 1.000 (ref) |  |
| Center 2 | 0.983 | 0.526 | 3.488 | 0.062 | 2.672 (0.953, 7.492) |  |
| Mechanical ventilation |  |  |  |  |  | 0.783 |
| No |  |  |  |  | 1.000 (ref) |  |
| Yes | 0.202 | 0.731 | 0.076 | 0.783 | 1.223 (0.292, 5.128) |  |
| Total length of hospital stay (days) | 0.018 | 0.038 | 0.229 | 0.633 | 1.019 (0.945, 1.098) | 0.633 |
| Duration of CPAP (days) | 0.036 | 0.036 | 0.981 | 0.322 | 1.037 (0.965, 1.113) | 0.322 |
| Gestational age at first oral feeding (weeks) | -0.067 | 0.269 | 0.061 | 0.805 | 0.936 (0.552, 1.586) | 0.805 |
| Time to regain birth weight (days) | 0.038 | 0.093 | 0.165 | 0.685 | 1.039 (0.865, 1.247) | 0.685 |
| Days with indwelling gastric tube (days) | -0.014 | 0.041 | 0.109 | 0.742 | 0.986 (0.910, 1.070) | 0.742 |
| Birth weight (g) | 0.001 | 0.001 | 0.921 | 0.337 | 1.001 (0.999, 1.003) | 0.337 |
| Gestational age at birth (weeks) | 0.496 | 0.281 | 3.121 | 0.077 | 1.643 (0.947, 2.849) | 0.077 |
| Frequency of breastfeeding or pumping within 48 h after delivery (times) | 0.011 | 0.098 | 0.013 | 0.909 | 1.011 (0.835, 1.225) | 0.909 |
| Depression score (points) | -0.145 | 0.095 | 2.365 | 0.124 | 0.865 (0.718, 1.041) | 0.124 |
| Anxiety score (points) | -0.083 | 0.034 | 5.843 | 0.016 | 0.921 (0.861, 0.984) | 0.016 |
| Maternal breastfeeding self-efficacy (points) | 0.023 | 0.031 | 0.567 | 0.451 | 1.023 (0.964, 1.087) | 0.451 |
| Paternal breastfeeding self-efficacy (points) | 0.052 | 0.023 | 4.941 | 0.026 | 1.053 (1.006, 1.102) | 0.026 |

Note: Any direct breastfeeding (exclusive or partial feeding at the breast) during the first 7 days after NICU discharge was the dependent variable (any DBF = 1, no DBF = 0). All variables examined in the univariate analysis were entered simultaneously, with study center retained as a fixed effect. β, regression coefficient; SE, standard error; aOR, adjusted odds ratio; CI, confidence interval; NMI, Neonatal Medical Index; DBF, direct breastfeeding; CPAP, continuous positive airway pressure. The reference category of each categorical variable is shown as 1.000 (ref), and an overall P value is given for each multi-level categorical variable. The variables with a Wald P < 0.05 in this full model were then retained in a single step and refitted to give the final adjusted model reported in Table 3.

**Supplementary Table 4. Comparison of baseline and care-practice characteristics between the two study centers (n = 421).**

| **Characteristic** | **Overall (n = 421)** | **Center 1 (n = 253)** | **Center 2 (n = 168)** | **Statistic** | **P** |
| --- | --- | --- | --- | --- | --- |
| Feeding outcome (any DBF vs. no DBF) |  |  |  | χ² = 5.904 | 0.015 |
| No DBF | 206 (49) | 136 (54) | 70 (42) |  |  |
| Any DBF | 215 (51) | 117 (46) | 98 (58) |  |  |
| Gestational age at birth (weeks) | 34.00 (33.00, 35.00) | 34.00 (33.00, 35.00) | 34.00 (33.00, 35.00) | Z = 0.126 | 0.899# |
| Birth weight (g) | 2130.00 (1800.00, 2345.00) | 2092.50 (1832.50, 2312.50) | 2181.00 (1776.00, 2460.00) | Z = -0.497 | 0.619# |
| Severity of illness |  |  |  | χ² = 6.552 | 0.088 |
| Grade 1 | 125 (30) | 80 (32) | 45 (27) |  |  |
| Grade 2 | 84 (20) | 47 (19) | 37 (22) |  |  |
| Grade 3 | 157 (37) | 86 (34) | 71 (42) |  |  |
| Grade ≥4 | 54 (13) | 39 (15) | 15 (9) |  |  |
| Oropharyngeal colostrum administration |  |  |  | χ² = 8.508 | 0.004 |
| None | 194 (46) | 131 (52) | 63 (38) |  |  |
| ≥1 session | 226 (54) | 121 (48) | 105 (62) |  |  |
| DBF during hospitalization |  |  |  | χ² = 0.478 | 0.489 |
| None | 230 (55) | 142 (56) | 88 (53) |  |  |
| ≥1 session | 190 (45) | 111 (44) | 79 (47) |  |  |
| Kangaroo mother care |  |  |  | χ² = 5.973 | 0.015 |
| None | 184 (44) | 123 (49) | 61 (37) |  |  |
| ≥1 session | 236 (56) | 130 (51) | 106 (63) |  |  |
| Admission to family-centered ward |  |  |  | χ² = 0.029 | 0.865 |
| Yes | 189 (45) | 113 (45) | 76 (46) |  |  |
| No | 231 (55) | 140 (55) | 91 (54) |  |  |
| Anxiety score (points) | 26.58±8.20 | 27.00±8.55 | 25.96±7.62 | t = 1.265 | 0.206† |
| Paternal breastfeeding self-efficacy (points) | 45.70±17.73 | 44.97±17.68 | 46.79±17.79 | t = 1.031 | 0.303† |

Note: Values are n (%) or median (interquartile range) or mean ± standard deviation. Percentages are rounded to the nearest whole number, and the denominator of each percentage is the number of participants in the relevant column (421 overall, 253 at center 1 and 168 at center 2). Categorical variables were compared using Pearson’s χ² test; #Mann–Whitney rank-sum test; †independent-samples t-test. NMI, Neonatal Medical Index; DBF, direct breastfeeding.

**Supplementary Table 5. Number of missing values for each variable, overall and by feeding group (n = 421).**

| **Variable** | **n with data** | **Missing, overall (n = 421)** | **Missing, no-DBF group (n = 206)** | **Missing, any-DBF group (n = 215)** |
| --- | --- | --- | --- | --- |
| Feeding outcome (any DBF vs. no DBF) | 421 | 0 (0.00%) | 0 (0.00%) | 0 (0.00%) |
| Total length of hospital stay (days) | 420 | 1 (0.24%) | 1 (0.49%) | 0 (0.00%) |
| Duration of CPAP (days) | 419 | 2 (0.48%) | 2 (0.97%) | 0 (0.00%) |
| Gestational age at first oral feeding (weeks) | 420 | 1 (0.24%) | 1 (0.49%) | 0 (0.00%) |
| Time to regain birth weight (days) | 420 | 1 (0.24%) | 1 (0.49%) | 0 (0.00%) |
| Days with indwelling gastric tube (days) | 420 | 1 (0.24%) | 1 (0.49%) | 0 (0.00%) |
| Birth weight (g) | 420 | 1 (0.24%) | 1 (0.49%) | 0 (0.00%) |
| Gestational age at birth (weeks) | 421 | 0 (0.00%) | 0 (0.00%) | 0 (0.00%) |
| Frequency of breastfeeding or pumping within 48 h after delivery (times) | 419 | 2 (0.48%) | 2 (0.97%) | 0 (0.00%) |
| Depression score (points) | 421 | 0 (0.00%) | 0 (0.00%) | 0 (0.00%) |
| Anxiety score (points) | 421 | 0 (0.00%) | 0 (0.00%) | 0 (0.00%) |
| Maternal breastfeeding self-efficacy (points) | 421 | 0 (0.00%) | 0 (0.00%) | 0 (0.00%) |
| Paternal breastfeeding self-efficacy (points) | 421 | 0 (0.00%) | 0 (0.00%) | 0 (0.00%) |
| Mode of delivery | 420 | 1 (0.24%) | 1 (0.49%) | 0 (0.00%) |
| Ventricular septal defect or patent ductus arteriosus | 407 | 14 (3.33%) | 12 (5.83%) | 2 (0.93%) |
| Oropharyngeal colostrum administration | 420 | 1 (0.24%) | 1 (0.49%) | 0 (0.00%) |
| Sex | 420 | 1 (0.24%) | 1 (0.49%) | 0 (0.00%) |
| Severity of illness | 420 | 1 (0.24%) | 1 (0.49%) | 0 (0.00%) |
| Admission to family-centered ward | 420 | 1 (0.24%) | 1 (0.49%) | 0 (0.00%) |
| DBF during hospitalization | 420 | 1 (0.24%) | 1 (0.49%) | 0 (0.00%) |
| Kangaroo mother care | 420 | 1 (0.24%) | 1 (0.49%) | 0 (0.00%) |
| Type of medical insurance | 420 | 1 (0.24%) | 1 (0.49%) | 0 (0.00%) |
| Educational level | 420 | 1 (0.24%) | 1 (0.49%) | 0 (0.00%) |
| Monthly household income (CNY) | 420 | 1 (0.24%) | 1 (0.49%) | 0 (0.00%) |
| Time to initiate milk expression after delivery | 420 | 1 (0.24%) | 1 (0.49%) | 0 (0.00%) |
| Breast pump use within 6 h after delivery | 420 | 1 (0.24%) | 1 (0.49%) | 0 (0.00%) |
| Breast massage within 24 h after delivery | 420 | 1 (0.24%) | 1 (0.49%) | 0 (0.00%) |
| Study center | 421 | 0 (0.00%) | 0 (0.00%) | 0 (0.00%) |
| Mechanical ventilation | 420 | 1 (0.24%) | 1 (0.49%) | 0 (0.00%) |

Note: Missing values were not imputed, and every analysis used all available data. The denominator of each percentage is the number of participants in the relevant column (421 overall, 206 in the no-DBF group and 215 in the any-DBF group). The number of infants with available data therefore differs between variables, and the denominator used for each variable in the univariate comparison is given in the note to Table 1. DBF, direct breastfeeding.

**Supplementary Table 6. Sensitivity analysis: final multivariable logistic regression model after excluding direct breastfeeding during hospitalization (n = 404).**

| **Variable** | **β** | **SE** | **Wald** | **P** | **aOR (95% CI)** | **Overall P** |
| --- | --- | --- | --- | --- | --- | --- |
| Intercept | -1.231 | 1.049 | 1.378 | 0.240 | 0.292 (0.037, 2.281) |  |
| Oropharyngeal colostrum administration |  |  |  |  |  | <0.001 |
| None |  |  |  |  | 1.000 (ref) |  |
| ≥1 session | 1.626 | 0.385 | 17.842 | <0.001 | 5.083 (2.390, 10.808) |  |
| Severity of illness |  |  |  |  |  | <0.001 |
| Grade 1 |  |  |  |  | 1.000 (ref) |  |
| Grade 2 | -0.754 | 0.550 | 1.876 | 0.171 | 0.471 (0.160, 1.384) |  |
| Grade 3 | -2.591 | 0.510 | 25.763 | <0.001 | 0.075 (0.028, 0.204) |  |
| Grade ≥4 | -3.607 | 0.671 | 28.867 | <0.001 | 0.027 (0.007, 0.101) |  |
| Educational level |  |  |  |  |  | <0.001 |
| Junior college or below |  |  |  |  | 1.000 (ref) |  |
| Bachelor's degree or above | 1.773 | 0.422 | 17.678 | <0.001 | 5.891 (2.577, 13.465) |  |
| Monthly household income |  |  |  |  |  | 0.005 |
| <10,000 CNY |  |  |  |  | 1.000 (ref) |  |
| 10,000-19,999 CNY | 1.153 | 0.463 | 6.213 | 0.013 | 3.169 (1.280, 7.850) |  |
| ≥20,000 CNY | 1.782 | 0.601 | 8.798 | 0.003 | 5.942 (1.830, 19.291) |  |
| Study center |  |  |  |  |  | 0.291 |
| Center 1 |  |  |  |  | 1.000 (ref) |  |
| Center 2 | 0.410 | 0.388 | 1.117 | 0.291 | 1.508 (0.704, 3.227) |  |
| Anxiety score | -0.106 | 0.030 | 12.772 | <0.001 | 0.900 (0.849, 0.953) | <0.001 |
| Paternal breastfeeding self-efficacy score | 0.072 | 0.012 | 37.575 | <0.001 | 1.074 (1.050, 1.099) | <0.001 |

Note: To examine whether the associations were robust to the conceptual and temporal proximity of DBF during hospitalization to the post-discharge outcome, the final-model procedure was repeated after excluding DBF during hospitalization: all 27 remaining variables were entered simultaneously into a full model, study center was retained as a fixed effect, and the variables with an overall Wald P < 0.05 were retained in a single step and refitted (n = 404). The retained variables were identical to those in the final model (Table 3 of the manuscript) except for the excluded variable, and no association changed direction or statistical significance: oropharyngeal colostrum administration changed from aOR 5.58 (95% CI 2.48–12.58) to 5.08 (2.39–10.81), anxiety score from 0.90 (0.85–0.96) to 0.90 (0.85–0.95), and paternal breastfeeding self-efficacy from 1.07 (1.04–1.10) to 1.07 (1.05–1.10). β, regression coefficient; SE, standard error; aOR, adjusted odds ratio; CI, confidence interval; DBF, direct breastfeeding. The reference category of each categorical variable is shown as 1.000 (ref), and an overall P value is given for each multi-level categorical variable.
